# The Protective Effects of *Pistacia Atlantica* Gum in a Rat Model of Aluminum Chloride-Induced Alzheimer’s Disease via Affecting BDNF and NF-kB

**DOI:** 10.5812/ijpr-142203

**Published:** 2024-06-22

**Authors:** Mohammad Mehdi Gravandi, Seyede Zahra Hosseini, Seyede Darya Alavi, Tayebeh Noori, Antoni Sureda, Roshanak Amirian, Mohammad Hosein Farzaei, Samira Shirooie

**Affiliations:** 1 Student Research Committee, Kermanshah University of Medical Sciences, Kermanshah, Iran; 2 Pharmaceutical Sciences Research Center, Health Institute, Kermanshah University of Medical Sciences, Kermanshah, Iran; 3 Research Group on Community Nutrition and Oxidative Stress (NUCOX) and Health Research Institute of Balearic Islands (IdISBa), University of Balearic Islands-IUNICS, Palma de Mallorca E-07122, Balearic Islands, Spain

**Keywords:** Alzheimer’s Disease, *Pistacia Atlantica*, NF-κB, BDNF, Rat

## Abstract

Alzheimer's disease (AD) is a neurodegenerative condition characterized by progressive cognitive deterioration, including deficits in memory and other cognitive functions. Oxidative stress and free radical damage play significant roles in its pathogenesis. This study aimed to investigate the potential anti-inflammatory and neuroprotective effects of *Pistacia atlantica* gum (administered at doses of 50 and 100 mg/kg for 14 days) in a rat model of AD induced by aluminum chloride (AlCl_3_). Behavioral changes were assessed using open field, passive avoidance, and elevated plus maze tests. Additionally, nitrite levels, nuclear factor-kappa B (NF-κB), brain-derived neurotrophic factor (BDNF), and immunostaining were evaluated. Administration of *P. atlantica* gum significantly increased step-through latency in the passive avoidance test (P < 0.01 and P < 0.001), enhanced mobility in the open field test (P < 0.01 and P < 0.001), and reduced anxiety-like behaviors in the elevated plus maze (P < 0.001) compared to the AlCl_3_ group. Treatment with the gum partially normalized the elevated levels of NF-κB and the decreased levels of BDNF caused by AlCl_3_ exposure. Our findings suggest that *P. atlantica* gum administration may alleviate oxidative stress, neuroinflammation, and cognitive impairment in AD rats.

## 1. Background

As the leading cause of dementia, Alzheimer's disease (AD) is a neurodegenerative disorder characterized by progressive impairment in behavioral and cognitive functions (1). The intracellular accumulation of neurofibrillary tangles (NFTs) and extracellular deposition of Amyloid-β (Aβ) plaques are the main pathophysiological hallmarks of AD (2). The clinical manifestation of AD is the gradual loss of memory and cognitive function, followed by neuropsychiatric symptoms such as periods of confusion, mood changes, delusions, and hallucinations that ultimately result in death (3). Approximately 60 - 70% of dementia patients suffer from AD, and it is estimated that dementia affects 55 million people worldwide (4-6). By 2050, this number is expected to surpass 152 million, with most of the growth occurring in low-income developing countries (5). The complex and multifactorial nature of AD represents the most difficult challenge in identifying an effective therapy capable of modifying the course of the disease and halting its progression (1). The presence of elevated levels of inflammatory markers and the identification of AD risk genes associated with innate immune functions suggest that neuroinflammation plays a significant role in the pathogenesis of AD (7, 8). Neuroinflammation is a chronic process that involves synaptic dysfunction, neuronal death, and inhibition of neurogenesis (8). Upon stimulation by Aβ plaques and NFTs, microglia and astrocytes become activated, migrate around plaques, and release neurotoxins and inflammatory molecules (8). The secretion of pro-inflammatory cytokines such as IL-1β, IL-6, IL-18, and tumor necrosis factor (TNF), as well as different chemokines like C-C motif chemokine ligand 1 (CCL1), CCL5, and C-X-C motif, along with the release of prostaglandins, nitric oxide, and reactive oxygen species (ROS), are associated with neurodegeneration at different stages of AD progression (8, 9). Therefore, considerable evidence suggests that targeting neuroinflammation is promising in AD drug discovery (5).

The current standard treatments for AD mainly focus on counteracting neurotransmitter imbalance. Acetylcholinesterase inhibitors (donepezil, rivastigmine, and galantamine) delay the cognitive decline in patients with AD by increasing acetylcholine levels and enhancing neuronal communication (10). While these medications relieve symptoms, they do not address the underlying pathology of the disease (11, 12). Conversely, new drugs tested in clinical trials as novel attempts to revive AD drug discovery have failed to demonstrate cognitive or clinical benefits (11, 12).

Brain-derived neurotrophic factor (BDNF) is a small protein belonging to the nerve growth factor (NGF) family, highly expressed in the mammalian brain (13). Brain-derived neurotrophic factor plays several prominent roles in the growth, development, differentiation, and regeneration of various types of neurons in the CNS. Additionally, it contributes to long-term potentiation and long-term depression, as well as learning and memory processes (14).

Certainly, nuclear factor-κB (NF-κB) is a well-known inflammatory transcription factor involved in neurodegeneration, consisting of NF-κB1 (p105/p50), NF-κB2 (p100/p52), RelA (p65), RelB, and c-Rel. The p65/p50 dimer, activated by IL-1β, Aβ peptide, or glutamate, induces proapoptotic genes, resulting in neuronal death (15).

Plant-derived resin oils are enriched sources of antioxidants and anti-inflammatory chemicals with potential therapeutic uses. One of the best examples of such plants is *Pistacia atlantica* Desf., a tree from the family Anacardiaceae that grows in the Zagrossian region. *P. atlantica* resin has been widely used in traditional medicine due to its manifold favorable impacts on biological activities, such as positive gastrointestinal effects on appetite, nausea, vomiting, and constipation, as well as beneficial neurological effects, such as nerve tonic in some pathological conditions, including Bell’s palsy, stroke, tetanus, seizure, tremor, and headache (16, 17). These beneficial activities presumably derive from the presence of phenolic compounds as well as monoterpenes and oxygenated sesquiterpenes (15-19). Numerous studies have evidenced the antioxidant and anti-inflammatory capacities of different extracts and essential oils of *P. atlantica* containing α-pinene, myrcene, limonene, β-pinene, and γ-terpineol, which may have pharmacological interest by reducing the production of reactive species and pro-inflammatory mediators via 2,2'-diphenyl-1-picrylhydrazyl (DPPH) radical scavenging, reducing ferric oxidation in the ferric reducing antioxidant power (FRAP) test, as well as decreasing lipid peroxidation in the TBARS assay (18-22). More specifically, various studies have shown the anti-inflammatory effects of essential oils from *P. atlantica* in vitro, as well as promoting wound healing in in vivo studies (23-25). Regarding the neuroprotective properties, in traditional Persian medicine, the essential oils of this species have been prescribed to promote memory (22). It has been depicted that some phenolic compounds such as methyl gallate and digalloylquinic have acetylcholinesterase (AChE) and anti-butyrylcholinesterase (BuChE) activity, a hopeful source for finding and expanding new anti-Alzheimer agents (26). The protective effects of *P. atlantica* extract (150 mg/kg/day, orally) were also observed in mercury-treated rats (2.5 mg/kg, once a week, i.p.), attenuating some of the harmful and toxic effects in the brain arising from mercury, such as reducing the activity of the antioxidant defense system through a substantial decline in the activity of catalase, glutathione peroxidase, glutathione-s-transferase, and superoxide dismutase acetylcholinesterase and elevation of the activity of lactate dehydrogenase (27).

## 2. Objectives

The aim of the present study was to evaluate the potential anti-inflammatory and neuroprotective effects of *P. atlantica* in a rat model of AD induced by aluminum chloride (AlCl_3_) via targeting NF-κB/BDNF.

## 3. Methods

This study was conducted following the standard instructions for working with animals of Kermanshah University of Medical Sciences, Iran (ethical number: IR.KUMS.AEC.1400.012). The study was also conducted in accordance with the policies for experimental and clinical studies outlined by Basic & Clinical Pharmacology & Toxicology (28).

### 3.1. Materials

Aluminum chloride, 99% pure, was provided by Sigma-Aldrich (St. Louis, MO). *P. atlantica*, containing α-pinene (42.9%) and β-pinene (13.2%) (29), was purchased from the Department of Pharmacognosy at the Faculty of Pharmacy at Kermanshah University of Medical Sciences in Iran.

### 3.2. Animals and Experimental Procedure

In this study, 32 adult male Wistar rats weighing between 200 - 230 g were purchased from Aftab Lorestan and housed in the central animal house at Kermanshah University of Medical Sciences. The animals were fed ad libitum and maintained under standard conditions (12-hour light/dark cycle, relative humidity of 60% ± 5%, and temperature of 24 ± 2ºC). Animal care strategies were carried out following the guidelines for the treatment and care of laboratory animals published by The Iranian National Institute of Health and approved by the Ethics Committee at Kermanshah University of Medical Sciences. All efforts were made to minimize mouse distress.

The rats were randomly divided into four groups, with each group containing eight rats (n = 8):

- Group 1: Control group consisting of normal rats treated intraperitoneally (i.p.) with saline for 14 days.

- Group 2: Animals receiving AlCl_3_ (100 mg/kg, i.p.) (30) for 14 days to induce memory dysfunction.

- Group 3: Animals receiving AlCl_3_ (i.p.) followed by oral administration of *P. atlantica* (50 mg/kg) (31) for 14 days.

- Group 4: Animals receiving AlCl_3_ (i.p.) followed by oral administration of *P. atlantica* (100 mg/kg) (31) for 14 days.

### 3.3. Open Field Test (OFT)

Following the initiation of AlCl_3_ treatment, an open field test (OFT) was conducted on the 8th day and repeated on the 14th day. The OFT is a fundamental sensorimotor test used to assess general activity in animal models of central nervous system disorders such as AD (32). The procedure was performed in a transparent plastic square box measuring 80 × 80 × 40 cm, allowing the animal to move freely within the box (33). The animals were observed for ten minutes in the center of the box, and data were collected on the following parameters: The number of squares traveled by the rats (line crossed), grooming behavior (hand licking), and rearing behavior (standing on two legs).

### 3.4. Elevated Plus Maze

The elevated plus-maze apparatus is a plus-shaped labyrinth comprising a central region, two opposing closed arms, and two opposing open arms. It serves as a behavioral assay for rodents to assess the anxiolytic effects of pharmacological agents (34, 35). Rats were placed in the open arm (on days 8 and 14), and it was determined whether the rats preferred the open arms over the closed arms. The time taken to transfer to the closed arm was measured.

### 3.5. Passive Avoidance Test

The passive avoidance test is utilized to assess learning and memory in animal models of CNS disorders. In this test, animals learn to avoid an environment in which an undesirable stimulus was previously delivered (36). The apparatus for the test comprises a chamber divided into a lighted compartment and a dark compartment, separated by a door. Rats inherently explore their surroundings, including dark and enclosed environments. Based on this natural behavior, the experiment was designed so that the rats were placed in a well-lit room on the training days (day 7), with the door open. The rats entered the dark chamber due to their preference for the dark environment; then, the door was closed, and an electric shock was immediately administered for one second. The time it takes the animal to move from the light side to the dark side of the apparatus was recorded as the initial transition latency (ITL). On the day of the experiment (days 8 and 14), the above procedure was repeated to assess the rats' cognitive abilities in recalling the previous day's electric shock experience and to determine whether the tendency to enter the darkroom had diminished. The time delay for the animal to enter the dark side during the memory retrieval phase (days 8 and 14) was designated as the memory criterion and recorded as step-through latency (STL) (37).

### 3.6. Histopathological Assessments

At the conclusion of the behavioral tests, all animals were euthanized (using 80 mg/kg ketamine + xylazine 10 mg/kg). The hippocampal tissue was carefully extracted and preserved in 200 mL of 4% paraformaldehyde and 0.1 M PBS. Tissue sections, 5 mm in thickness, were prepared using a scalpel and stained with hematoxylin and eosin (H&E) stain. Histopathological alterations were examined using a light microscope, and data were collected at a magnification of 400.

### 3.7. Nuclear Factor Kappa B p65 and Brain-Derived Neurotrophic Factor Immunostaining

Immunostaining analyses of the hippocampus were performed using the affinity purification method to evaluate the expression levels of NF-κB and BDNF (38). Sections were deparaffinized (2 x 5 min in TBS plus 0.03% Triton X-100). The NF-κB test probe and BDNF test probe were incubated with 0.5 μg/mL anti-NF-kB p65 antibody (ab16502) and anti-BDNF antibody (ab108319) (Turbo: At 37ºC for 4 hours, overnight at 4ºC). Then, they were washed (3 × 5 min in TBS plus 0.03% Triton X-100) and incubated with 0.2 mg/mL secondary antibody (Goat Anti-Rabbit IgG (H + L) (FITC) (E-AB-1014)) in a dark room at 37°C for 90 min. Afterwards, they were washed three times with DAPI (Sigma-D9542) and for 20 min with PBS. Next, sections were dehydrated in ethanol and xylene, then covered with a fluorescent mounting medium. The sections were observed under an Olympus BX50 fluorescence microscope (39).

### 3.8. Statistics

Data analysis was performed using GraphPad Prism 9. One-way analysis of variance (ANOVA) followed by Tukey’s post hoc test was used to determine the differences between groups. Results were presented as mean ± SEM, and P < 0.05 was considered statistically significant.

## 4. Results

### 4.1. Behavioral Tests

#### 4.1.1. Open Field Test

**Figure 1.**
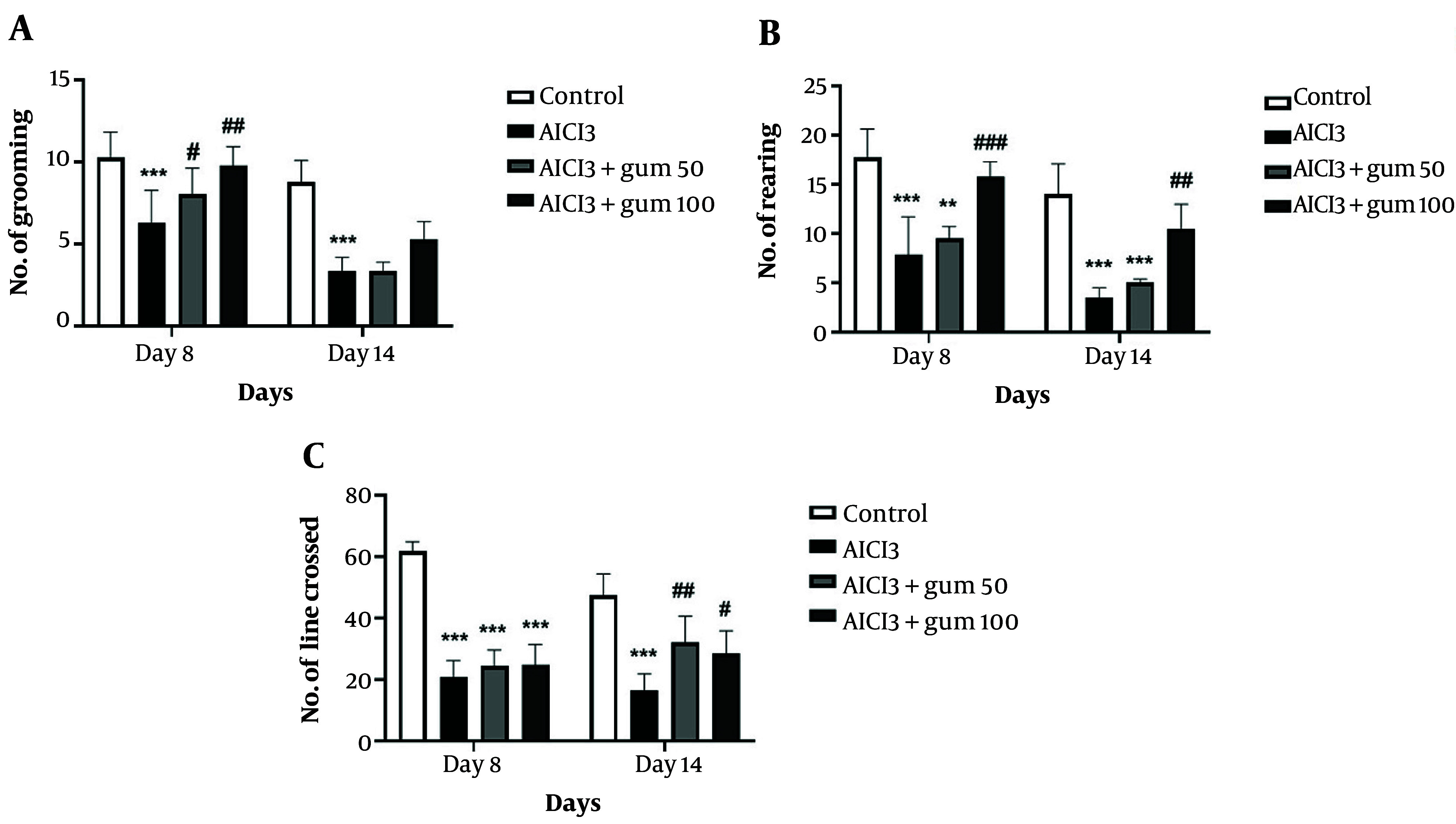
The results of the open field test in animals exposed to AlCl_3_ and treated with *P. atlantica* gum. A, the number of grooming acts; B, number of rearing acts; C, number of lines crossed. AlCl_3_, aluminium chloride 100 mg/kg i.p; AlCl_3_ + gum 50, *P. atlantica* gum 50 mg/kg treated-AlCl_3_ group; AlCl_3_ + gum 100, *P. atlantica* gum 100 mg/kg treated-AlCl_3_ group. #P < 0.05; ##P < 0.01; ###P < 0.001 compared with the AlCl_3_ group; **P < 0.01; ***P < 0.001 compared with the control group.

The general levels of activity, locomotor activity, and exploration behaviors in a new environment determined by the open field test are shown in Figure 1. The group treated with AlCl_3_ showed significantly less grooming, rearing, and crossing lines compared to the control group (P < 0.001). Administration of both doses of *P. atlantica* (50 and 100 mg/kg) induced a progressive recovery in grooming and rearing at the two time points analyzed, while for the crossed lines, the changes are less clear and only evident at 14 days.

#### 4.1.2. Elevated Plus Maze

**Figure 2.**
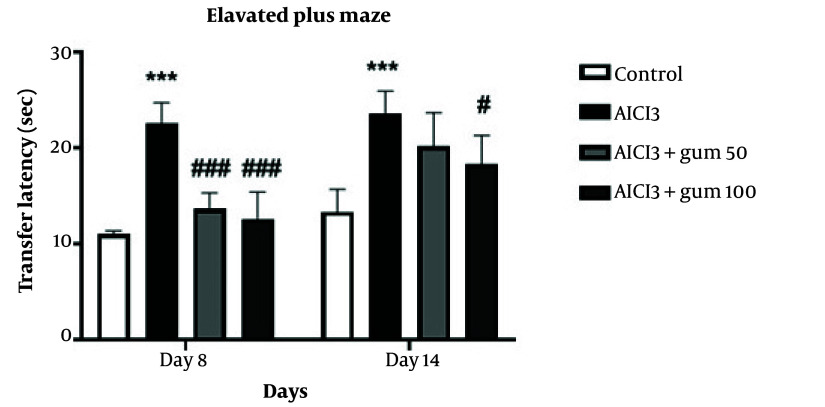
Anxiety-like behaviors in the elevated-plus-maze in animals exposed to AlCl_3_ and treated with *P. atlantica* gum. AlCl_3_, aluminium chloride 100 mg/kg i.p; AlCl_3_ + gum 50, *P. atlantica* gum treated-AlCl_3_ group; AlCl_3_ + gum 100, *P. atlantica* gum 100 mg/kg treated-AlCl_3_ group. #P < 0.05, and ###P < 0.001 compared with the AlCl_3_ group; ***P < 0.001 compared with the control group.

The effects of *P. atlantica* on the transfer latency of rats treated with AlCl_3_ in the elevated plus-maze are presented in Figure 2. The transfer latency of the AlCl_3_-treated group was significantly increased compared to the control group (P < 0.001). Treatment with both concentrations of *P. atlantica* significantly decreased transfer latency compared to the AlCl_3_ group at 8 days (P < 0.001), while at 14 days, it was only significant for the 100 mg/kg concentration (P < 0.05).

#### 4.1.3. Passive Avoidance Test

**Figure 3.**
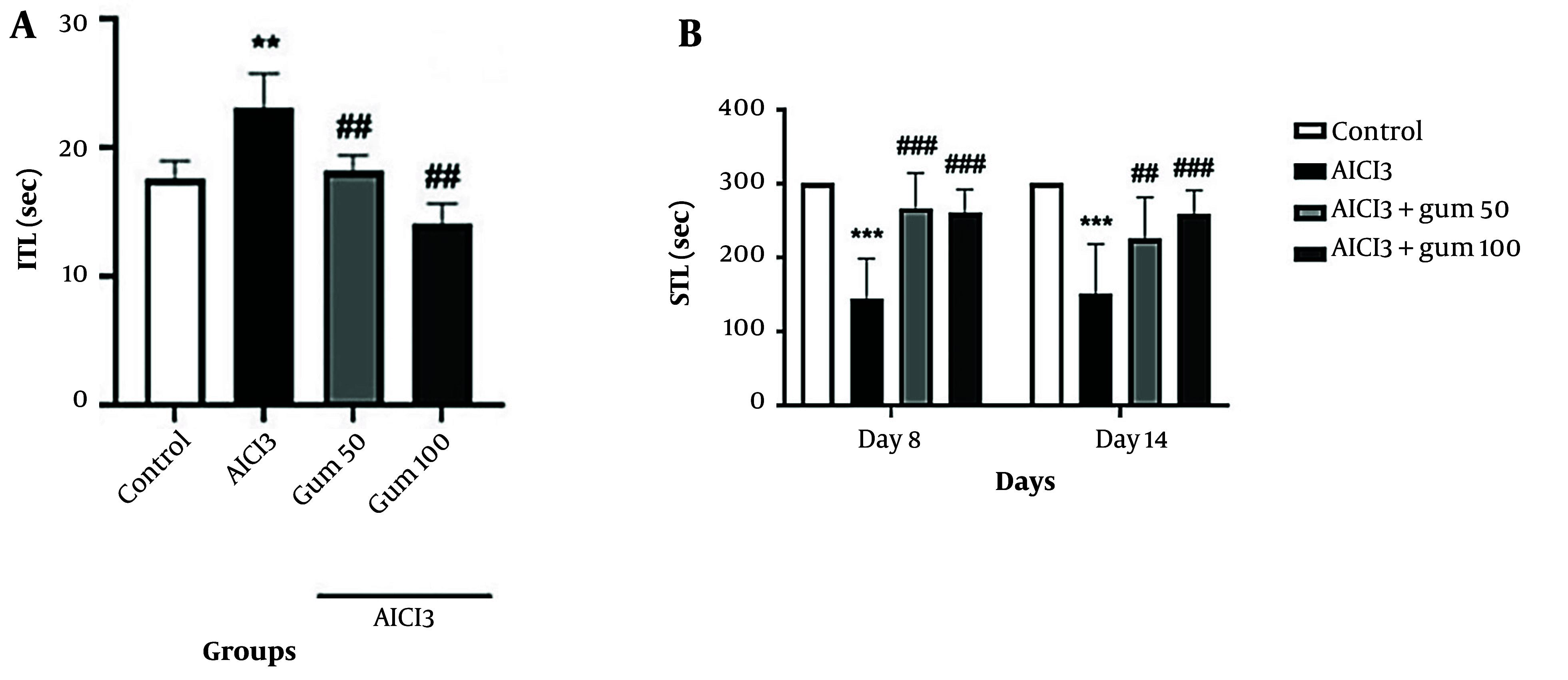
Passive avoidance test in animals exposed to AlCl_3_ and treated with *P. atlantica* gum. A, ITL (initial transfer latency); B, comparison of STL (step-through latency) during a Passive Avoidance test. AlCl_3_, aluminum chloride 100 mg/kg i.p; AlCl_3_ + gum 50, *P. atlantica* gum 50 mg/kg treated-AlCl_3_ group; AlCl_3_ + gum 100, *P. atlantica* gum 100 mg/kg treated-AlCl_3_ group. ##P < 0.01; ###P < 0.001 compared with the AlCl_3_ group; **P < 0.01; ***P < 0.001 compared with the control group.

The results of memory and learning abilities of rats determined with the passive avoidance test are presented in Figure 3. Treatment with AlCl_3_ significantly increased ITL (P < 0.01) and decreased STL (P < 0.001) compared with the control group. *Pistacia atlantica*-treated groups performed the test better in both short-term memory and long-term memory compared to the AlCl_3_ group (P < 0.01 and P < 0.001), with the group receiving 100 mg/kg showing a better response in STL after 14 days.

#### 4.1.4. Histopathological Studies

**Figure 4.**
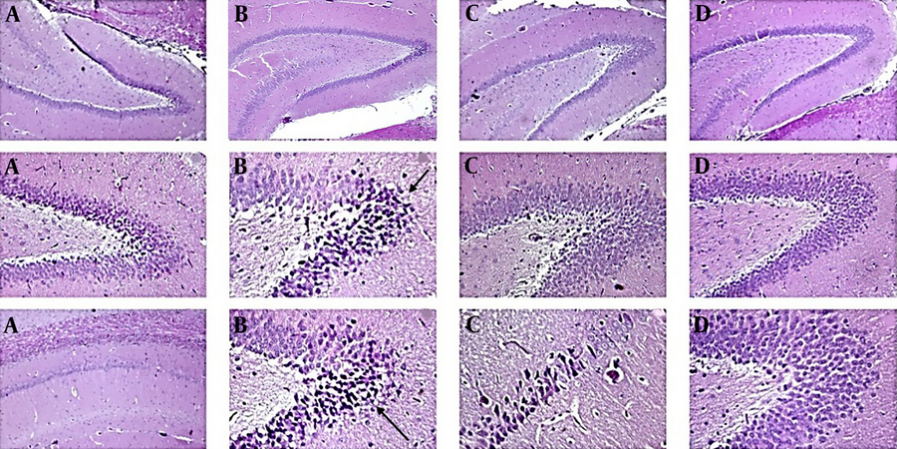
Photomicrographs of the hippocampus stained with hematoxylin and eosin (*400). A, control group; B, AlCl3 100 mg/kg i.p.; C, P. atlantica gum 50 mg/kg treated-AlCl3 group; D, P. atlantica gum 100 mg/kg treated-AlCl3 group.

The control group (Figure 4A) presented normal hippocampus layers and typical morphological characteristics, such as granule cells with intact nuclear details and an undamaged hilar area. In the AlCl_3_ group (Figure 4B), apoptotic cells (dark) could be observed locally in some areas of the hippocampus. An increase in the perivascular space, an abnormal appearance in some gray matter cells (cerebral cortex), abnormal collection and compression in cortical arteries and veins (even capillaries), a relative decrease in the number of cells in the gray matter layers (cerebral cortex), and the hippocampus could be observed. Additionally, the presence of dark apoptotic cells in the neuroglial population and the analysis of cellular excesses in the deep areas adjacent to the hippocampus could be observed.

In the group treated with *P. atlantica* 50 mg/kg (Figure 4C), a relative decrease in the changes caused by AlCl_3_ was observed, including a decrease in apoptotic dark cells and a relative increase in the neuronal layer of the hippocampus, a decrease in the perivascular space, and a relative normalization of the appearance of the vessels (arteries and veins). However, dark cells are still present in small areas of the hippocampus, although their intensity is lower than in the AlCl_3_ group. The appearance of tissue sections of the cerebral cortex and hippocampus in the 100 mg/kg treated group was very similar to the normal group (Figure 4D). The thickness of the neuronal layer of the hippocampus was increased compared to the AlCl_3_ group, and the neurons are normal. Dark cells are very few, and the perivascular space was similar to the normal group. The vascular layer adjacent to the hippocampus was also normal, and the network of cellular extravasations resembled that of the control group. The cortical layer of the brain (gray matter) looks similar to the control group in terms of cell density and the appearance of extracellular background cells.

#### 4.1.5. Brain-Derived Neurotrophic Factor levels

**Figure 5.**
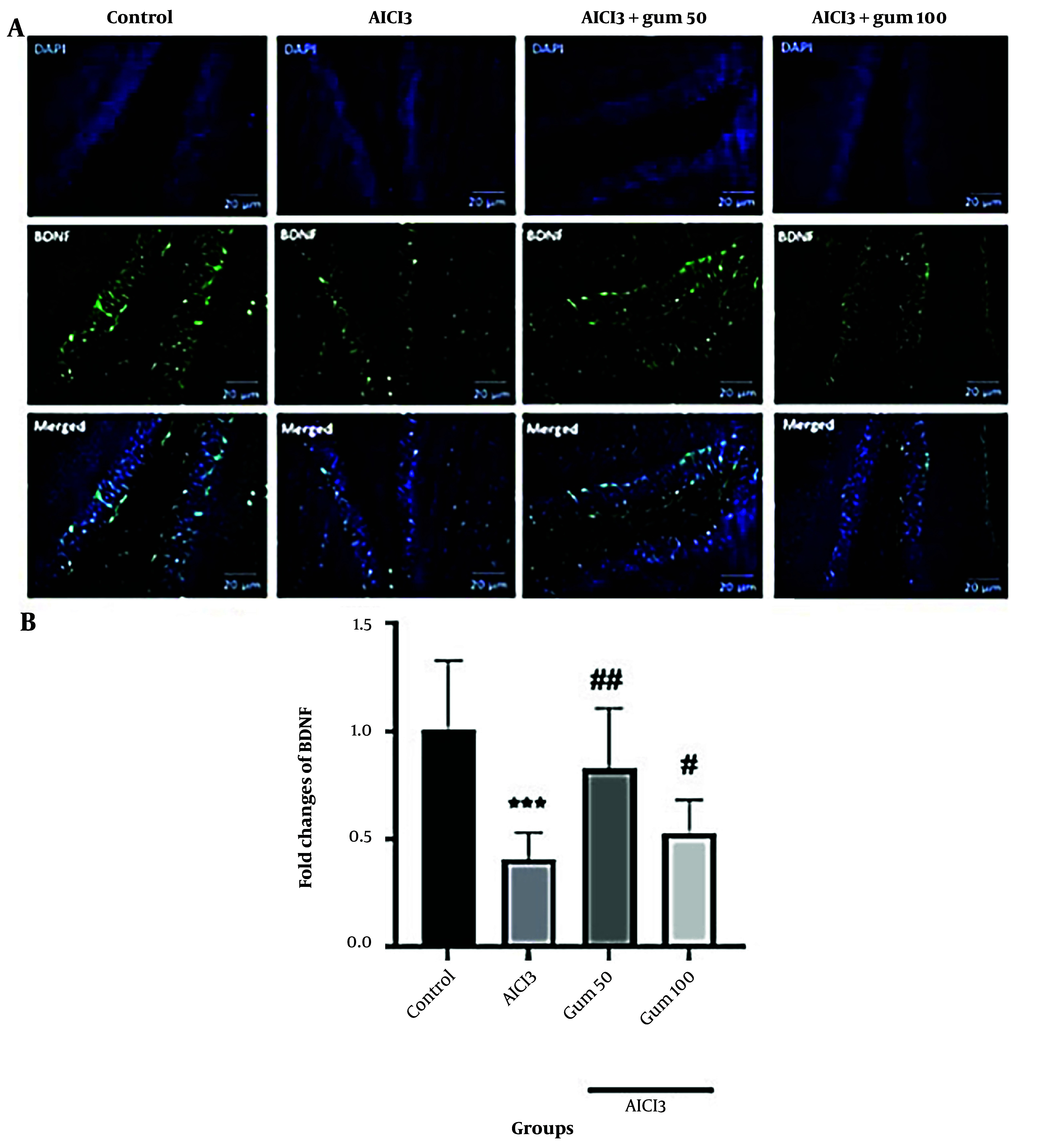
Levels of brain-derived neurotrophic factor (BDNF) in hippocampi by immunofluorescence analysis. A, microphotographs show the co-localization of BDNF (green)-positive cells with DAPI (blue) in the hippocampi of animals, B, semi-quantitative assay of active BDNF. AlCl_3_, aluminum chloride 100 mg/kg i.p; AlCl_3_ + gum 50, *P. atlantica* gum 50 mg/kg treated-AlCl_3_ group; AlCl_3_ + gum 100, *P. atlantica* gum 100 mg/kg treated-AlCl_3_ group. #P < 0.05; ## P < 0.01 compared with the AlCl_3_ group; ***P < 0.001 compared with the control group.

According to immunofluorescence analysis, a reduction in the levels of BDNF (P < 0.001) was observed in the AlCl_3_ group compared to the control group (Figure 5). The administration of *P. atlantica* partially recovered the BDNF levels, mainly in the 50 mg/kg group (P < 0.01).

#### 4.1.6. Nuclear Factor Kappa B Levels

**Figure 6.**
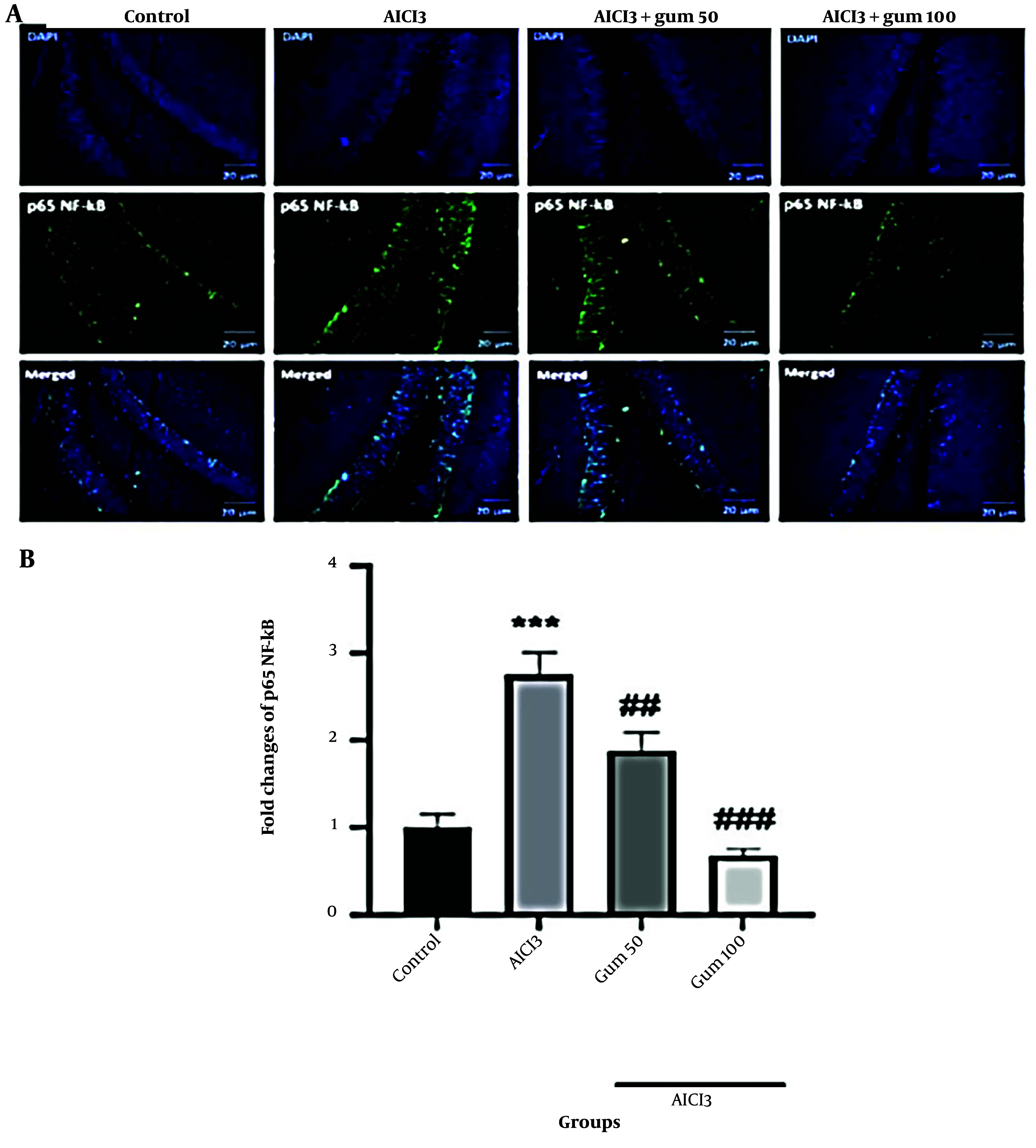
Levels of nuclear factor-kappa B (NF-κB) p65 in the brain tissues by immunofluorescence analysis. A, microphotographs show the co-localization of NF-κB p65 (green)-positive cells with DAPI (blue) in the hippocampi of animals; B, semi-quantitative assay of active NF-κB p65. AlCl_3_, aluminum chloride 100 mg/kg i.p; AlCl_3_ + gum 50, *P. atlantica* gum 50 mg/kg treated-AlCl_3_ group; AlCl_3_ + gum 100, *P. atlantica* gum 100 mg/kg treated-AlCl_3_ group. ##P < 0.01; ###P < 0.001 compared with the AlCl_3_ group; ***P < 0.001 compared with the control group.

The protein levels of p65 NF-κB were significantly increased in the hippocampus of the AlCl_3_ group (P < 0.001) compared to the control group (Figure 6). The administration of *P. atlantica* progressively decreased the levels of p65 NF-κB protein (P < 0.01 and P < 0.001).

## 5. Discussion

Alzheimer's disease is a progressive disorder characterized by memory loss and cognitive decline (40). The amyloid beta (Aβ) cascade theory, tau theory, inflammation theory, cholinergic theory, and oxidative theory are among the most recognized hypotheses proposed to explain AD (41, 42). An important aspect of AD pathogenesis related to neuroinflammation is the accumulation of Aβ in the brain (43). Elevated levels of reactive oxygen species (ROS), increased microglial activation, cytokine release, and activated NF-κB all contribute to the neuroinflammatory process in AD (44). Nuclear factor-kappa B, as a key transcription factor, modulates the expression of several genes encoding proteins involved in immune and inflammatory responses (45). Brain-derived neurotrophic factor, a crucial neurotrophin for synaptic development and flexibility, shows impaired signaling in AD brains and is associated with tau phosphorylation, Aβ accumulation, neuroinflammation, and neuronal apoptosis (46). Indeed, altered BDNF signaling in animal models of AD exacerbates age-related memory impairment, while increases in its levels have beneficial effects on learning and memory (47, 48).

Currently, there are no effective treatments for AD; existing medications only slow down disease progression. Moreover, routinely used drugs (Donepezil, Rivastigmine, Galantamine, Tacrine, etc.) often have significant side effects, such as hepatotoxicity, underscoring the need to develop new drugs with minimal toxic effects (42, 48). Various natural compounds have been investigated for their potential anti-neuroinflammatory activity, acting through mechanisms such as microglia activation suppression, restriction of pro-inflammatory cytokine production, NF-κB suppression, and p38 mitogen‑activated protein kinase (MAPK) activation (49). Alkaloids, polyphenols, terpenes, and carotenoids are among the natural products showing anti-neuroinflammatory potential. Flavonoids and other polyphenolic substances, in particular, exhibit anti-inflammatory properties by reducing pro-inflammatory mediators and suppressing NF-κB and p38 MAPK pathways (50, 51). Flavonoids, due to their suppressive effects on pro-inflammatory transcription factors and activation of antioxidant/anti-inflammatory transcription factors, are considered a significant subgroup for reducing neuroinflammation in AD (51).

The wild pistachio, or *P. atlantica*, which grows in Iran, Turkey, Iraq, and Saudi Arabia, has been widely used in ancient medicine to treat various conditions, including upper abdominal discomfort, dyspepsia, and peptic ulcers (16). Through phytochemical investigations, various beneficial substances such as phenolic compounds, terpenes, fatty acids, tocopherols, and phytosterols have been identified (16). Previous studies have highlighted the high antioxidant content of *P. atlantica* leaves, suggesting potential protection against oxidative damage (42). Extracts of *P. atlantica* have demonstrated potent acetylcholinesterase (AChE) inhibitory, antioxidant, and antiproliferative effects (24, 26, 52-55). Moreover, several articles have discussed the anti-Alzheimer effects of *P. atlantica* (56-58). A study by Ben Ahmed et al. illustrated the potential of *P. atlantica* galls as a source for novel anti-AD substances, identifying metabolites in *P. atlantica* gall extracts that may contribute to anticholinesterase activity (26). Nuzzo et al.indicated that consuming pistachios with a high-fat diet prevented the negative effects of the diet on neurons and improved metabolic parameters, including oxidative stress, apoptosis, and mitochondrial dysfunction in mice (59). Additionally, the bicyclic monoterpene α-pinene, found in *P. atlantica*, has been shown to possess anxiolytic properties and improve locomotor activities (24, 60, 61). Its mechanism of action is associated with upregulation of BDNF mRNA expression in the hippocampal regions of rats following α-pinene inhalation (24, 61).

This study demonstrates the effectiveness of *P. atlantica* in treating and recovering rats with AD induced by AlCl_3_. The elevated plus maze, open field, and passive avoidance behavioral tests were used to assess behavioral alterations induced by AlCl_3_ and the protective effects of *P. atlantica*. The elevated plus maze test measures anxiety-related behavior (35). Aluminum chloride treatment increased the time taken to transition from the open arm to the closed arm, while *P. atlantica* reversed this increase.

Open field test is used to evaluate the level of motor and cognitive activities. The frequency of line crossings, rearing, and grooming is used to measure rat performance during the test period (62). In this study, the administration of *P. atlantica* improved the performance of the rats compared to the AlCl_3_ group. Similarly, in the passive avoidance test, the latency was more extended in the gum groups than in the AlCl_3_ group, which is related to better quality of memory.

Brain-derived neurotrophic factor is a critical factor in neuronal survival and memory and has been linked to the etiology of AD, with decreased BDNF levels found in the disease (63). It is a neurotrophin that plays a significant role in neuronal survival and growth and participates in the development and flexibility of synapses, which is essential for learning and memory (46, 64). The present results evidenced a decrease in BDNF protein levels in the hippocampus after AlCl_3_ administration, suggesting the role of this neurotrophin in AD. Moreover, the administration of *P. atlantica* was capable of partially recovering the levels of BDNF.

Alzheimer's disease is characterized by plaque deposits of the Aβ peptide and the neurofibrillary tangles of the microtubule-binding protein tau (65). Previous data have shown that the neurotoxic Aβ is a powerful stimulator of the transcription factor NF-κB in primary neurons (66, 67). An important aspect of controlling inflammatory reactions is the NF-κB pathway (36). The levels of NF-κB were increased in the AlCl3 group, which supports the findings that the NF-κB pathway is involved in this disorder. Consistent with the observed reduction of NF-κB after *P. atlantica* administration, previous studies have also shown that some phytochemicals such as morin, thymol, and thymoquinone reduce the levels of NF-κB in a model of AD induced by AlCl_3_ (68). Immunohistochemical analysis showed that AlCl_3_ causes the development of AD by triggering an increase in NF-κB and a decrease in BDNF. These data also suggest that consumption of *P. atlantica* reverses this effect.

From a histopathological standpoint, compared to the AlCl_3_ group, the groups treated with the gum had thicker neuronal layers in the hippocampus, and the neurons were normal. The perivascular space was identical to that of the normal group, and there were relatively few dark cells, which were occasionally visible as tiny local islands. The network of cellular extravasations was comparable to that of the normal group, and the vascular layer next to the hippocampus was also normal. Regarding cell density and the presence of extracellular background cells, the cortical layer of the brain (sometimes referred to as gray matter) resembled that of the normal group. Generally, the observation of the histological results further supports the improvement of AD.

### 5.1. Conclusions

The current study revealed the protective properties of *P. atlantica* and the damaging effects of AlCl_3_ consumption in an in vivo model of AD. The results of behavioral tests evidenced that *P. atlantica* could improve cognitive dysfunction. The inhibition of the NF-kB pathway and the induction of BDNF are possible mechanisms involved in the neuroprotective action of *P. atlantica*. Treatment with *P. atlantica* could also lessen oxidative stress by lowering the inflammatory reaction linked to the NF-kB pathway. These findings support the idea that *P. atlantica* might provide a potential treatment option for further studies on neurodegenerative diseases like AD.

## Data Availability

The dataset presented in the study is available on request from the corresponding author during submission or after publication.
